# Lipoxin A4 inhibits microglial activation and reduces neuroinflammation and neuropathic pain after spinal cord hemisection

**DOI:** 10.1186/s12974-016-0540-8

**Published:** 2016-04-08

**Authors:** Alessandra Cadete Martini, Temugin Berta, Stefânia Forner, Gang Chen, Allisson Freire Bento, Ru-Rong Ji, Giles Alexander Rae

**Affiliations:** Department of Pharmacology, Centro de Ciências Biológicas, Universidade Federal de Santa Catarina, Florianópolis, Santa Catarina 88040-970 Brazil; Departments of Anesthesiology and Neurobiology, Duke University Medical Center, Durham, NC 27710 USA; Centro de Inovação e Ensaios Pré-Clínicos-CIEnP, Florianópolis, Santa Catarina 88056-000 Brazil; Present address: Institute for Memory Impairments and Neurological Disorders, University of California, Irvine, CA 92697-4545 USA; Present address: Pain Research Center, Department of Anesthesiology, University of Cincinnati Medical Center, Cincinnati, OH 45267 USA

**Keywords:** Lipoxin, Microglia, Neuroinflammation, Chronic pain, Spinal cord injury

## Abstract

**Background:**

Spinal cord injury (SCI) is a severe neurological disorder with many disabling consequences, including persistent neuropathic pain, which develops in about 40 % of SCI patients and is induced and sustained by excessive and uncontrolled spinal neuroinflammation. Here, we have evaluated the effects of lipoxin A4 (LXA4), a member of a unique class of endogenous lipid mediators with both anti-inflammatory and analgesic properties, on spinal neuroinflammation and chronic pain in an experimental model of SCI.

**Methods:**

Spinal hemisection at T10 was carried out in adult male CD1 mice and Wistar rats. To test if LXA4 can reduce neuroinflammation and neuropathic pain, each animal received two intrathecal injections of LXA4 (300 pmol) or vehicle at 4 and 24 h after SCI. Sensitivity to mechanical stimulation of the hind paws was evaluated using von Frey monofilaments, and neuroinflammation was tested by measuring the mRNA and/or protein expression levels of glial markers and cytokines in the spinal cord samples after SCI. Also, microglia cultures prepared from murine cortical tissue were used to assess the direct effects of LXA4 on microglial activation and release of pro-inflammatory TNF-α.

**Results:**

LXA4 treatment caused significant reductions in the intensity of mechanical pain hypersensitivity and spinal expression levels of microglial markers and pro-inflammatory cytokines induced by SCI, when compared to rodents receiving control vehicle injections. Notably, the increased expressions of the microglial marker IBA-1 and of the pro-inflammatory cytokine TNF-α were the most affected by the LXA4 treatment. Furthermore, cortical microglial cultures expressed ALX/FPR2 receptors for LXA4 and displayed potentially anti-inflammatory responses upon challenge with LXA4.

**Conclusions:**

Collectively, our results suggest that LXA4 can effectively modulate microglial activation and TNF-α release through ALX/FPR2 receptors, ultimately reducing neuropathic pain in rodents after spinal cord hemisection. The dual anti-inflammatory and analgesic properties of LXA4, allied to its endogenous nature and safety profile, may render this lipid mediator as new therapeutic approach for treating various neuroinflammatory disorders and chronic pain with only limited side effects.

**Electronic supplementary material:**

The online version of this article (doi:10.1186/s12974-016-0540-8) contains supplementary material, which is available to authorized users.

## Background

Spinal cord injury (SCI) is a highly debilitating condition with a worldwide incidence that ranges from 40 to 80 cases per million people per year [1]. SCI is generally associated with a wide array of comorbidities that typically require lifelong therapy and rehabilitative care. Thus, SCI translates not only into enormous personal and financial losses for the patient but also into substantial burdens to national economies and health-care systems.

Pain is a severe problem for many SCI patients. It is estimated that 40 to 50 % of patients with SCI develop neuropathic pain within the first year after injury, a chronic pain condition resulting from nerve damage [2, 3]. Neuropathic pain after SCI is characterized by spontaneous persistent pain and evoked pain, such as mechanical allodynia (pain evoked by normally non-noxious tactile stimuli) and thermal hyperalgesia (an increased response to noxious thermal stimuli) at/or below the level of injury [4]. Unfortunately, clinical management of neuropathic pain is often ineffective or inadequate, mostly focusing on the modulation of the neuronal activity by targeting neuronal sodium and calcium channels or NMDA and GABA receptors [5]. However, in recent years, it has become evident that the development of neuropathic pain involves not only neuronal pathways but also components of the immune system and glial cells, such as astrocytes and microglia, that mount a local form of inflammation called neuroinflammation [6, 7].

SCI is marked by an extensive neuroinflammation that is characterized by early activation of microglia and astrocytes which can release chemokines and cytokines to enhance the infiltration of peripheral leukocytes into the spinal cord through the damaged blood-spinal cord barrier [8]. SCI pathology is reduced, and spontaneous recovery of neurological function (motor, sensory, and autonomic) is improved when the activation of glial cells and/or the recruitment of leukocytes are controlled [9]. Increasing evidence suggests that targeting neuroinflammation may also offer new opportunities for better management of neuropathic pain [10–12].

Lipoxins and resolvins are endogenous lipid mediators that contribute to control the inflammatory response and allow inflamed tissues to return to homeostasis once the need for inflammation is over [13, 14]. They are potent anti-inflammatory, pro-resolution, and analgesic mediators acting in the picomolar to nanomolar dose range, which is a desirable characteristic for drug development. Lipoxins and resolvins can attenuate neuroinflammation as well as inflammatory and neuropathic pain [15–18]. Due to the robust neuroinflammation and severe neuropathic pain that characterize SCI, we hypothesized that these lipid mediators may represent a novel strategy to control both excessive neuroinflammation and pain after SCI.

Lipoxin A4 (LXA4) is an eicosanoid generated from arachidonic acid via sequential actions of lipoxygenases that appears to act at both temporal and spatially distinct sites from other eicosanoids produced during the course of an inflammatory response, and that, alongside resolvins, stimulates natural resolution of the process [14]. Furthermore, stable analogs of LXA4 exist on the market and have shown potent anti-inflammatory and pro-resolution actions in vitro and in vivo [19]. Intrathecal LXA4 injections have been shown to quell pain in different animal models of chronic pain [18, 20, 21]. In particular, it was observed that LXA4 reduced inflammatory pain through activation of ALX/FPR2 receptors and blocking of MAPKs signaling in spinal cord astrocytes [15]. However, the unique role of LXA4 in neuropathic pain and its control of microglial signaling are virtually unknown. Therefore, the present study was designed to explore the possible anti-inflammatory and analgesic effects of LXA4 after spinal cord hemisection, a common rodent model of SCI.

## Methods

Experiments were performed on male CD1 mice and Wistar rats. The Institutional Animal Care and Use Committee of Duke University and the Ethics Committee on Animal Use of the Universidade Federal de Santa Catarina approved all animal procedures and experimental protocols.

### Animal surgery and drug administration

Spinal cord injury by left-side hemisection was performed as described previously [22], with some modifications. Under general anesthesia with 2–3 % isoflurane in oxygen, a longitudinal incision was made and a laminectomy was performed at two vertebral segments, T9–T10. The spinal cord was then hemisected at T10 on the left side by placing a 28-gauge needle dorsiventrally at the midline of the cord and pulling it laterally to ensure the completeness of the hemisection. Subsequently, the fascia, musculature, and skin were sutured.

### Intrathecal administration of drugs

**Fig. 1:**
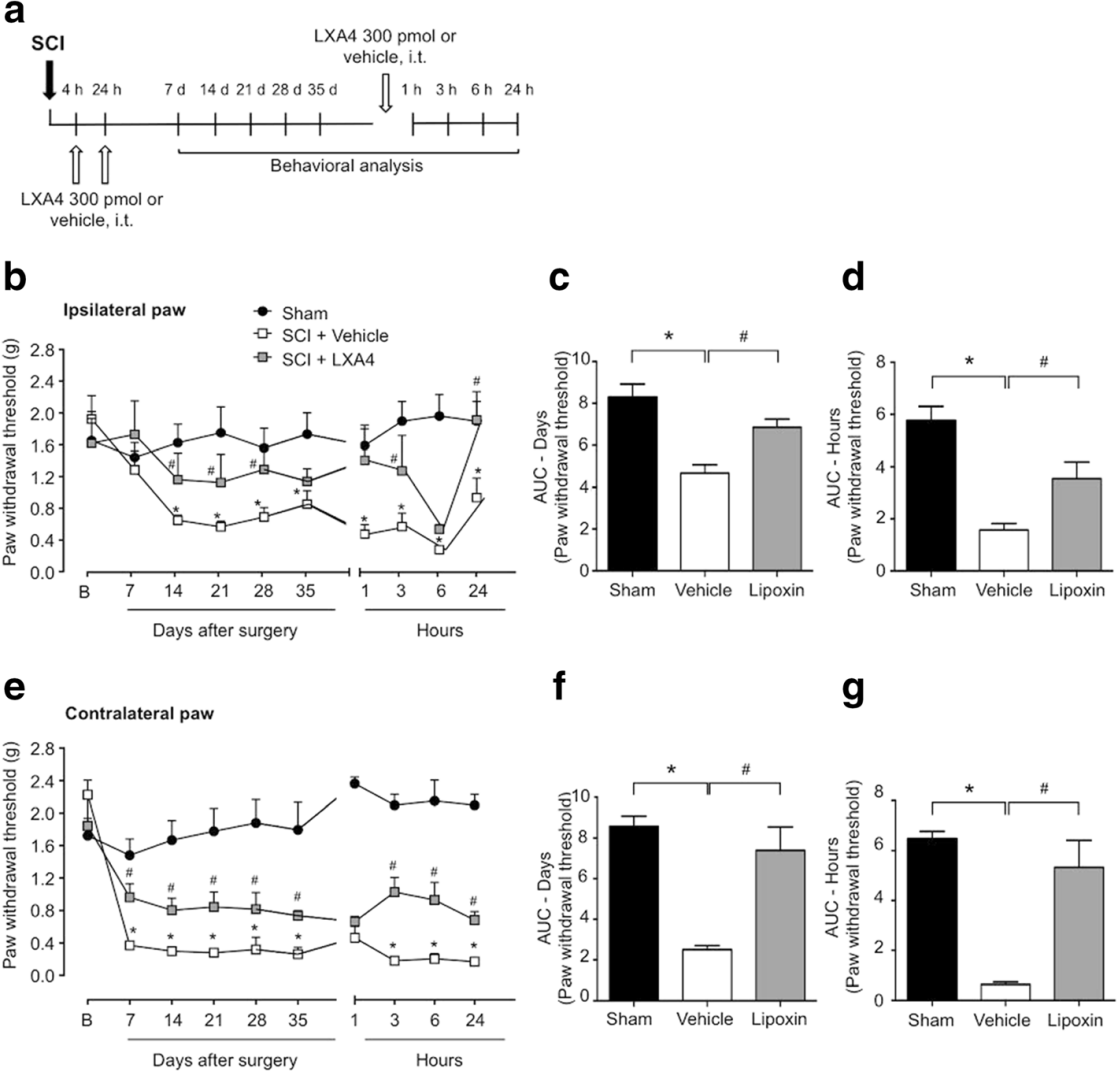
Lipoxin A4 attenuates mechanical allodynia induced by spinal cord hemisection in mice. **a** Treatment scheme for intrathecal injections of lipoxin A4 or vehicle and behavioral analyses. **b**, **e** Following SCI, adult mice develop mechanical allodynia in both ipsilateral and contralateral hindpaws. Fifty percent paw withdrawal thresholds are increased with LXA4 administration at 4 and 24 h, compared to vehicle-treated group. An additional intrathecal bolus injection of 300 pmol of LXA4 at 35 days still attenuated mechanical allodynia in both ipsilateral and contralateral paws from 3 h up to 24 h. **c**, **f**–**d**, **g** The area under the curve depicting variations of 50 % paw withdrawal thresholds in time are presented for treatment evaluation up to 35 days and after the bolus injection. Results are presented as mean ± SEM. *Asterisk* denotes *p* < 0.05 when comparing to sham-operated group and *number sign* denotes *p* < 0.05 when comparing to vehicle-treated group (two-way ANOVA followed by Bonferroni; *n* = 6 mice/group)

Intrathecal injections were performed under brief isoflurane anesthesia (2.7 %) by spinal cord puncture with a 28^1/2^-G needle connected to a 1-mL insulin syringe. The needle was inserted perpendicularly between the L4 and L5 levels of the spinal cord, and injections were given in a fixed volume of 10 μL. Lipoxin A4 (Cayman Chemicals Co.) was prepared in 0.5 % ethanol in sterile PBS and administered intrathecally 4 and 24 h after SCI surgery. A different group of mice also received a new injection of LXA4 or vehicle on the 35th day after surgery, according to Fig. 1a. ALX/FPR2 receptor short-interfering RNA (siRNA) or scrambled control (OriGene Technologies Inc.) were mixed with the transfection reagent polyethyleneimine (PEI, Fermentas), dissolved in 5 % glucose in RNase-free water [17]. The protocol for siRNA administration included two injections: one given at 72 h before surgery and a second injection given in combination with LXA4 at 4 h after surgery. At 24 h after surgery (20 h after completion of siRNA treatment), mice received another injection of LXA4 alone.

### Behavioral analysis

In mice, the mechanical thresholds for 50 % paw withdrawal were assessed by testing each animal’s reactions to successive application of multiple caliber von Frey hairs (vFh) to either hindpaw, using the up-down method [23]. Rats were evaluated through the estimation of the frequency of paw withdrawal responses to ten consecutive applications of a von Frey hair exerting 15*g* of force, applied perpendicularly to the ventral surface of each hindpaw at 30-s intervals. Mice mechanical sensitivity was assessed before surgery for a baseline estimate and on the 7th, 14th, 21st, 28th, and 35th days after surgery and/or treatment with LXA4 or vehicle. On the 35th day, animals received a new injection of LXA4 and were evaluated at 1, 3, 6, and 24 h post-treatment. For the siRNA-treated mice, behavioral analysis was performed on the seventh day after surgery. Rats’ mechanical sensitivity was assessed before surgery for a baseline estimate and on the 2nd, 7th, 14th, 21st, and 28th days after surgery and/or treatment with LXA4 or vehicle.

### Quantitative real-time RT-PCR (qPCR)

Spinal cord samples were collected 7 days after SCI or sham surgery. Total RNA was extracted using Direct-zol™ RNA MiniPrep Kit (Zymo Research Corporation) and reverse-transcribed using the *iScript cDNA Synthesis*® (Bio-Rad), following the respective protocols provided with the kits. Specific primers including the housekeeping control GAPDH were purchased from Sigma-Aldrich (Table 1). Gene-specific mRNA analysis was performed using CFX96 Real-Time system (Bio-Rad), and the relative quantities of mRNAs were calculated using the comparative Ct method [24, 25].

**Table 1:** Specific primers used for quantitative real-time RT-PCR (qPCR)

	Forward primers	Reverse primers	Accession no.
GAPDH	TTGATGGCAACAATCTCCAC	CGTCCCGTAGACAAAATGGT	NM_001001303
FPR2	CACAGGAACCGAAGAGTGTAAG	CACCATTGAGAGGATCCACAG	NM_008039
GFAP	GAATCGCTGGAGGAGGAGAT	GCCACTGCCTCGTATTGAGT	NM_010277
IBA1	GGACAGACTGCCAGCCTAAG	GACGGCAGATCCTCATCATT	NM_019467
P2RY12	TTTCAGATCCGCAGTAAATCCAA	GGCTCCCAGTTTAGCATCACTA	NM_016928
TNF-α	CCCCAAAGGGATGAGAAGTT	CACTTGGTGGTTTGCTACGA	NM_013693
IL-6	TCCATCCAGTTGCCTTCTTGG	CCACGATTTCCCAGAGAACATG	NM_031168
iNOS	GGAGTGACGGCAAACATGACT	TAGCCAGCGTACCGGATGA	NM_010927
TGFβ	CCACCTGCAAGACCATCGAC	CTGGCGAGCCTTAGTTTGGAC	NM_011577
IL-10	AGCCGGGAAGACAATAACTG	GGAGTCGGTTAGCAGTATGTTG	NM_010548

### PCR and gel electrophoresis

The spinal cord, cultured microglia, and astrocytes cDNA were tested for the presence of the ALX/FPR2 receptor. Primers for ALX/FPR2 (forward TATAGTGAGAGCCAAGTA, reverse AATGAGAGCAATCAAGAA) were used in PCR reactions containing sample cDNA, Taq RED Master Mix, 2.0X (Apex) 0.2 mM of each primers. A standard PCR protocol was run in a T100™ Thermal Cycler (Bio-Rad), and amplified products were displayed on a SYBR Safe DNA gel stain (Invitrogen) 1 % agarose gel.

### Primary cultures of microglia and astrocytes

Astrocyte and microglia cultures were prepared from cerebral cortexes of 2-day-old postnatal mice, as previously described [26]. Briefly, tissues were transferred to ice-cold Hank’s buffer, meninges were carefully removed, and tissues were triturated and collected by centrifugation. The cell pellets were dissociated and suspended in a medium containing 10 % fetal bovine serum in DMEM. After trituration and filtration, cells were plated into T75 flasks and cultured at 37 °C with 5 % CO_2_/95 % air. Medium was replaced every 3–4 days. Confluence was achieved after 2 weeks, and microglia started to grow over the astrocytes after 3 weeks. For preparing microglia cultures, the mixed glial cells were shaken for 4 h, and the floating cells (microglial pool) were collected and cultured at a density of 2.5 × 10^5^ cells/ml. This method resulted in >95 % purity of astrocytes or microglia. Before performing experiments with astrocytes, we added 0.15 mM dibutyryl cAMP (Sigma-Aldrich) to the culture for 2–3 days to induce astrocytic differentiation [27].

For analyzing TNF-α and p-p38 expression, microglia culture medium was replaced by Opti-MEM medium (Life Technologies, MA, EUA), containing 1 % penicillin/streptomycin, and cells were incubated with LXA4 (10 or 100 nM) for 30 min prior to IFN-γ (20 ng/mL) for 3 h.

### Western blot

Cells were collected by using RIPA buffer (Thermo Fisher Scientific), protein concentrations were determined by BCA Protein Assay (Pierce), and 20 μg of proteins was loaded for each lane and separated by SDS-PAGE gel (4–15 %; Bio-Rad). After the transfer, the blots were incubated overnight at 4 °C with polyclonal antibody against p-p38 (1:1000, Cell Signaling) or GAPDH for loading control (1:10000, Millipore). These blots were further incubated with HRP-conjugated secondary antibody, developed in ECL solution (Pierce), and the chemiluminescence was revealed by Bio-Rad ChemiDoc XRS for 1–5 min. Specific bands were evaluated by apparent molecular sizes. The intensity of the selected bands was analyzed using NIH Image J software.

### Enzyme-linked immusorbent array

Culture medium (Opti-MEM, Life Technologies) was collected after treatment, and protein concentration was determined by BCA Protein Assay (Pierce). TNF-α mouse ELISA kit was purchased from R&D Systems (R&D Systems, MN, USA). For each assay, 50 μL of culture medium was used and the standard curve was included in each experiment.

Rat spinal cord T9 to T11 segments were collected 2, 4, and 7 days after SCI, and protein concentration was determined by NanoDrop 1100 (Nanodrop Technologies, Wilmington, USA). TNF-α, IL-β, IL-6, and IL-10 rat ELISA kits were also purchased from R&D Systems.

### Immunofluorescence

For the spinal cord IF, mice were deeply anesthetized with isoflurane and perfused through the ascending aorta with PBS, followed by 4 % paraformaldehyde with 1.5 % picric acid in 0.16 M phosphate buffer. After the perfusion, the T9 to T11 spinal cord segments were removed and post-fixed in the same fixative overnight. Spinal cord sections (30 μm) were cut in a cryostat, and the sections were first blocked with 2 % goat serum for 1 h at room temperature. The sections were then incubated overnight at 4 °C with the primary antibodies against Iba-1 (1:1000; Wako Pure Chemical Industries) and GFAP (1:1000; Millipore). The sections were then incubated for 1 h at room temperature with cyanine 3 (Cy3)- or FITC-conjugated secondary antibodies (1:400; Jackson ImmunoResearch). DAPI (Vector laboratories) was used to stain cell nuclei. The stained and mounted sections were examined with a Nikon fluorescence microscope, and images were captured with a CCD SPOT camera.

Microglia cells were fixed in paraformaldehyde 4 % with 0.1 % picric acid for 20 min and then washed three times with PBS. Sections were incubated at room temperature for 30 min in PBS-10 % goat serum (Jackson ImmunoResearch) with 2 % bovine albumin (Sigma-Aldrich) and 0.4 % Triton-X100 (Sigma-Aldrich). Primary ALX/FPR2/FPRL1 antibody (1:200; Novus biologicals) was diluted in PBS-5 % goat serum (Sigma-Aldrich) with 1 % bovine albumin (Sigma-Aldrich) and 0.2 % Triton-X100 and incubated overnight at 4 °C. Corresponding anti-rabbit Cy3 (Jackson ImmunoResearch) was diluted in PBS containing 2 % goat serum (Sigma-Aldrich), 0.4 % bovine albumin (Sigma-Aldrich), and 0.08 % Triton-X100 and incubated at room temperature for 1 h for secondary detection. DAPI (Life Technologies) was used as counterstaining.

### Statistical analysis

All data are expressed as mean ± SEM, unless otherwise stated. The statistical significance between the groups was assessed using one or two-way ANOVA followed by the Bonferroni test or by Student’s paired or unpaired *t* tests, as appropriate. The criterion for statistical significance was *p* ≤ 0.05. All analyses were done with GraphPad Prism® 6 software (GraphPad Software Inc., San Diego, CA, USA).

## Results

### Lipoxin A4 exerts an antiallodynic effect in mice and rats after SCI

To investigate if LXA4 blocks pain associated with SCI, we used the spinal cord thoracic hemisection rodent model of SCI [22]. Typically, this model results in bilateral persistent below-level pain in mice and rats, including increase in mechanical allodynia (Fig. 1 and Additional file 1: Figure S1, respectively). Two intrathecal injections of 300 pmol LXA4, given at 4 and 24 h after SCI (Fig. 1a), exerted antiallodynic effects in both mice (Fig. 1b, c, e, f) and rats (Additional file 1: Figure S1A–D). In mice, the 50 % paw withdrawal thresholds was significantly increased by LXA4 treatment at 7 days after SCI in the contralateral paw only and bilaterally at 14 days (ipsilateral paw *F*(2,8) = 12.41, *p* = 0.0035; contralateral paw *F*(2,8) = 13.80, *p* = 0.0026), as compared with the vehicle-treated mice (Fig. 1b, c), and this attenuation of allodynia by LXA4 was maintained for up to 35 days after SCI (Fig. 1e, f). LXA4 also promoted a similar bilateral antiallodynic effect from 7 to 28 days after SCI in rats (Additional file 1: Figure S1).

Because lipoxins are sensitive to metabolic inactivation by dehydrogenation [14] and neuropathic pain persisted for at least 5 weeks, we tested whether an additional single bolus intrathecal injection of 300 pmol of LXA4 at 35 days after SCI (Fig. 1a) could still exert antiallodynic effects in mice. Interestingly, this late LXA4 treatment significantly attenuated mechanical allodynia in both the ipsilateral (*F*(2,8) = 13.23, *p* = 0.0029) and contralateral paws (*F*(2,8) = 107.2, *p* < 0.0001) from 3 h up to 24 h in mice (Fig. 1b, d, e, g).

### Lipoxin A4 exerts its antiallodynic effect through ALX/FPR2 receptors

**Fig. 2:**
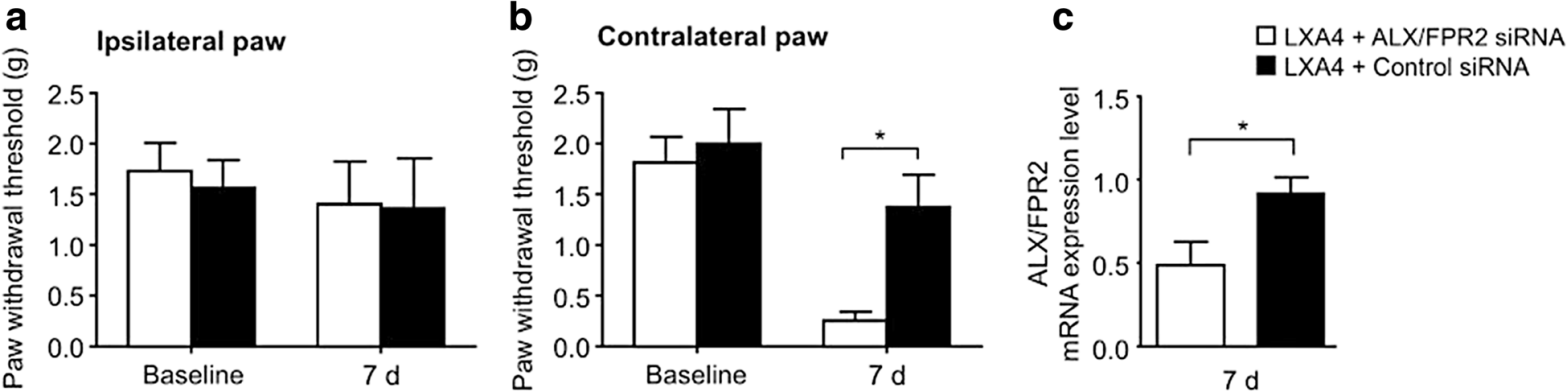
ALX/FPR2 receptor gene knockdown with intrathecal siRNA treatment impairs the analgesic effect of LXA4 after SCI in mice. **a** No differences were observed on the 7th day post-surgery with the siRNA administration in the ipsilateral paw. **b** Gene knockdown suppresses the antihyperalgesic effects of LXA4 observed in the contralateral paw. **c** On the 7th day after surgery, there is a significant decrease on ALX/FPR2 receptor mRNA levels in the spinal cord of animals injected with the specific siRNA. Results are presented as mean ± SEM. *Asterisk* denotes *p* < 0.05 when comparing to control-siRNA-treated group (two-way ANOVA followed by Bonferroni, *n* = 6 mice/group)

LXA4 binds with high affinity to a G protein-coupled ALX/FPR2 receptor [28, 29]. To test whether the antiallodynic effect of LXA4 was mediated through the ALX/FPR2 receptor, we used intrathecal injections of ALX/FPR2 siRNA at 2 days before and on the same day of surgery to significantly knockdown the expression of ALX/FPR2 receptors by ~50 % in the spinal cord, as compared to the control non-target siRNA (Fig. 2c). We tested the antiallodynic effect of LXA4 at 7 days after SCI, since the siRNA effect has a tendency to wear off after 1 week [30]. Treatment with ALX/FPR2 receptor siRNA did not cause any change to the mechanical sensitivity of the ipsilateral paw (Fig. 2a), which is consistent with the absence of allodynia observed at this time point (Fig. 1b). However, SCI-induced mechanical allodynia in the contralateral paw was unaffected by LXA4 treatment in mice receiving ALX/FPR2 siRNA, whereas it was significantly reduced by LXA4 treatment in mice receiving control non-target siRNA (Fig. 2b). These results suggest that the analgesic effects of intrathecal LXA4 were mediated, at least in part, through spinal ALX/FPR2 receptors.

### LXA4 decreases the SCI-induced reactivity of microglial cells, but not astrocytes

**Fig. 3:**
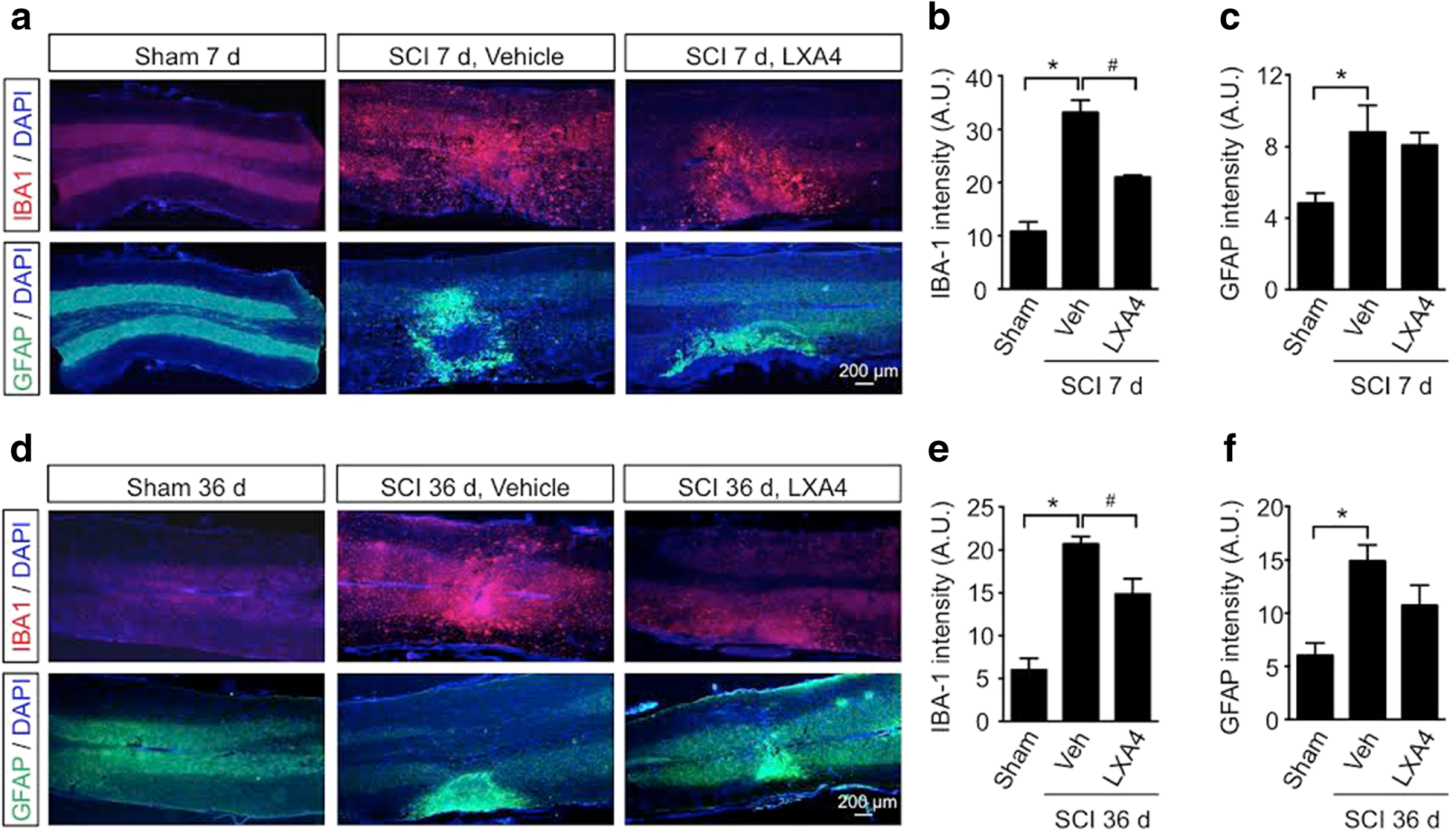
LXA4 attenuates the SCI-induced microglial reactivity in the spinal cord. Lipoxin A4 reduces the SCI-induced increase in Iba-1 expression in the spinal cord at 7 and 36 days after surgery. Staining for Iba-1 (*red*, **a**), GFAP (*green*, **b**), and DAPI (*blue*) in thoracic spinal cord from sham, vehicle-treated SCI, or LXA4-treated SCI mice. Scale 200 μm. Iba-1 staining was enhanced at 7 (**a**, **b**) and 36 days (**d**, **e**) after SCI and was reduced by LXA4 treatment. GFAP expression was significantly upregulated by SCI at 7 (**a**–**c**) and 36 days (**d**–**f**) but was not changed following LXA4 administration. Results shown in graphs are presented as mean ± SEM. *Asterisk* denotes *p* < 0.05 when comparing to sham-operated group. *Number sign* denotes *p* < 0.05 when comparing with vehicle-treated group (Student’s *t* test, *n* = 6 mice/group)

Because LXA4 has been shown to target glial cells [31–33] and these cells play important roles in pain hypersensitivity [34–36], we next sought to determine if the observed antiallodynic effects of the lipid mediator could be mediated via alterations in reactivity of spinal microglia and/or astrocytes in mice after SCI. We used the most common glial markers, including IBA-1 for microglia and GFAP for astrocytes, to reveal SCI-induced changes in spinal glial reactivity and their susceptibility to treatment with LXA4. We performed IBA-1 and GFAP immunohistochemistry on thoracic spinal cord sections from mice 7 and 36 days post SCI (Fig. 3a, d, respectively). We found that SCI induced remarkable changes in both microglia and astrocytes, as revealed by increases in staining intensity of IBA-1 and GFAP in the spinal cord ipsilateral to injury at 7 and 36 days (Fig. 3b, c, e, f). Compared to the vehicle-treated SCI group, IBA-1 staining in the LXA4-treated SCI group was significantly decreased by 36.7 ± 1.3 % at 7 days and 27.0 ± 9.4 % at 36 days of (Fig. 3b, e), but GFAP staining was not modified (Fig. 3c, f). In line with our present results, a recent study demonstrated the expression of ALX/FPR2 receptors in both microglia and astrocytes and an anti-inflammatory effect of LXA4 in CNS [37].

### Lipoxin A4 reduces SCI-induced expression of microglial markers and synthesis of pro-inflammatory mediators in the spinal cord

**Fig. 4:**
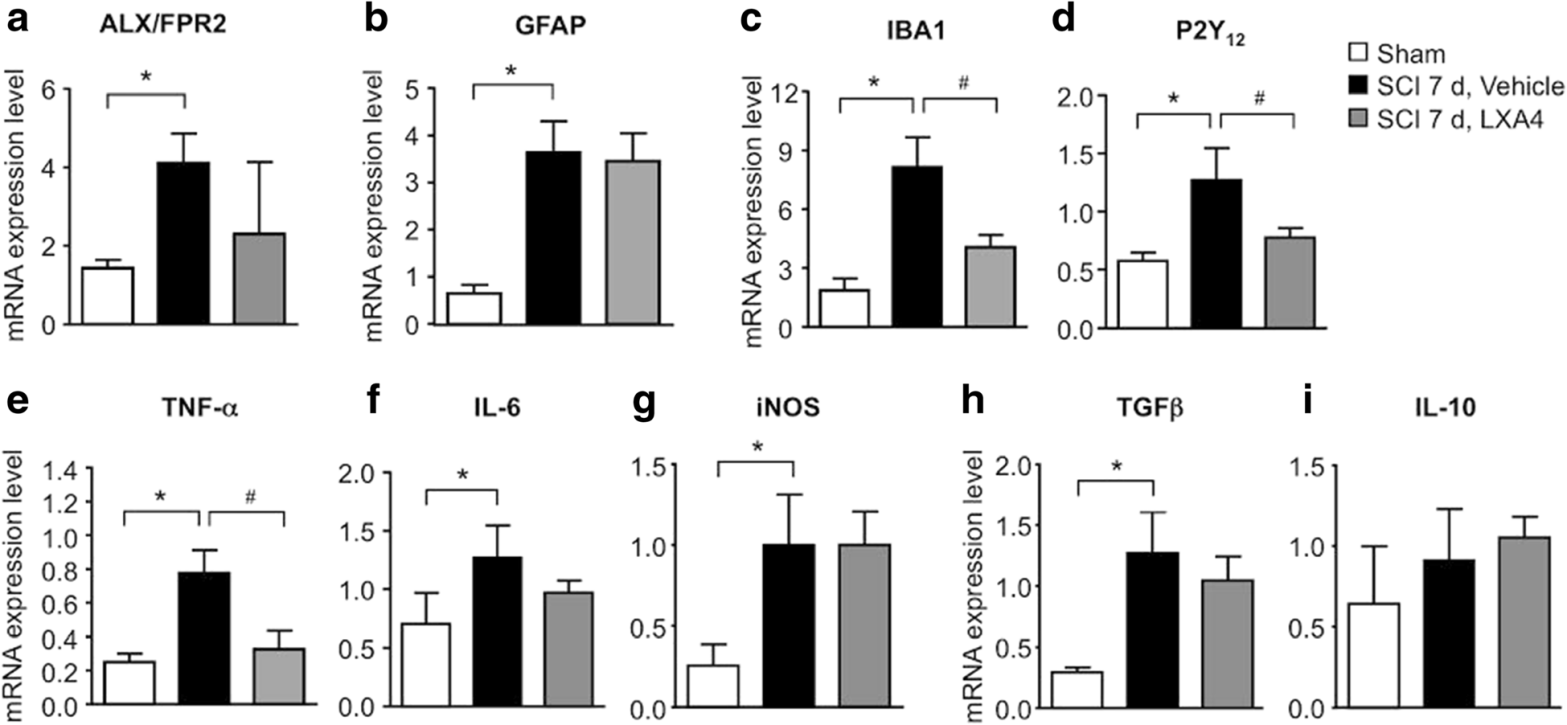
Lipoxin A4 reduces the expression of microglial markers and TNF-α. **a**–**h** Spinal cord injury promotes a significant upregulation of ALX/FPR2 receptors, GFAP, IBA-1, P2Y_12_, TNF-α, IL-6, iNOS, and TGF-β mRNA levels 7 days post-surgery, comparing to sham-operated mice. **c**–**e** LXA4 significantly reduced IBA-1, P2Y_12_, and TNF-α mRNA levels. Data is expressed as mean ± SEM. *Asterisk* denotes *p* < 0.05 when comparing to sham-operated group. *Number sign* denotes *p* < 0.05 when comparing with vehicle-treated group (one-way ANOVA followed by Bonferroni; *n* = 4 mice/group)

To confirm the cellular effect and identify the molecular target of LXA4 in the CNS, we screened the expression of well-known glial markers and inflammatory mediators 7 days after SCI by qPCR. First, we verified that ALX/FPR2 was similarly expressed in thoracic spinal cords from mice receiving a vehicle control or a LXA4 injection, supporting comparable drug-receptor interactions in both conditions (Fig. 4a). Furthermore, we confirmed the potential effect of LXA4 on microglia but not astrocytes. Consistently, the mRNA expression level of the astrocytic marker GAFP was unaffected by LXA4 treatment (Fig. 4b), whereas microglial markers IBA-1 and P2Y12 were significantly decreased in mice treated with LXA4 when compared to vehicle-treated mice (Fig. 4c). Microglia generally respond to injury by releasing, for instance, cytokines, nitric oxide, and growth factors [38–41]. Quantitative real-time RT-PCR analyses of TNF-α, IL-6, iNOS, IL-10, and TGFβ in mice showed that LXA4 treatment significantly decreased the transcriptional expression of TNF-α compared to the control vehicle treatment (Fig. 4d). Protein analyses of thoracic spinal cords from rats not only confirmed the effect of LXA4 on TNF-α expression levels but also revealed a significant decrease in expression levels of IL-6 and IL-1β, suggesting predominant post-transcriptional mechanisms for LXA4 action (Additional file 2: Figure S2A–C). Notably, LXA4 treatment did not affect the expression levels of the anti-inflammatory cytokine IL-10 (Fig. 4e and Additional file 2: Figure S2D) and of the growth factor TGFβ (Fig. 4e). Altogether, these results suggest SCI-induced increases in the microglial reactivity and synthesis of pro-inflammatory cytokines near the injury is partially blocked by LXA4 treatment through a potential interaction of LXA4 with microglial cells.

### LXA4 inhibits TNF-α release in microglial cultures

**Fig. 5:**
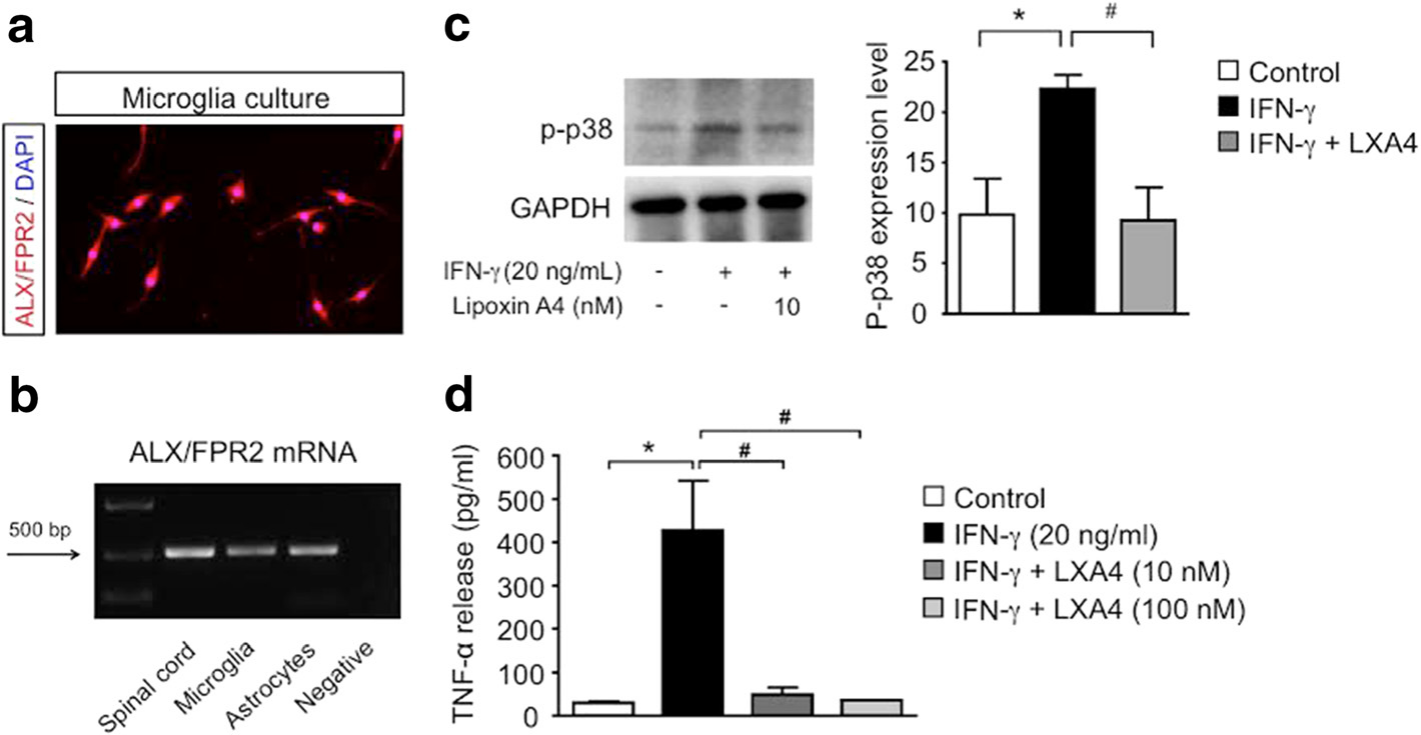
Lipoxin A4 attenuates microglial activation in primary cultures. **a** Microglia cells stained for DAPI (*blue*) and ALX/FPR2 receptor (*red*). Scale 50 μm. **b** Representative image of ALX/FPR2 mRNA expression on the spinal cord and microglia and astrocytes cultures. **c** LXA4 reduced p-p38 increase induced by IFN-γ. **d** IFN-γ induces a significant increase in TNF-α release, which is reduced by both concentrations of LXA4. Data is expressed as mean ± SEM. *Asterisk* denotes *p* < 0.05 when comparing to control. *Number sign* denotes *p* < 0.05 when comparing with vehicle (one-way ANOVA followed by Bonferroni; *n* = 4 cultures)

To test a direct effect of LXA4 on microglial activation, we sought to examine the effect of LXA4 in primary microglia cultures. First, we verified the expression of the ALX/FPR2 receptor in microglia culture by immunofluorescence (Fig. 5a) and by RT-PCR (Fig. 5b). Next, we activated microglia culture with 20 ng/ml of IFN-γ for 3 h, since this cytokine can potently activate microglia and also play a role in spinal pain processing [42]. To determine whether LXA4 affects microglia activation, we examined the phosphorylation of the p-38 mitogen-activating kinase (p-p38), which is a critical step of microglia activation in various neuropathic pain models [43–47]. There was a significant increase of p-p38 in microglia subjected to IFN-γ, but this increase was abrogated in cultures treated with 10 nM of LXA4 (Fig. 5c). Next, we tested if LXA4 treatment would abrogate IFN-γ-induced TNF-α release. Similar to our in vivo results (Fig. 4d and Additional file 2: Figure S2A), IFN-γ-induced microglial TNF-α release was significantly decreased by LXA4 treatment, when compared to the vehicle-treated controls (Fig. 5d). These in vivo results indicate a direct effect of LXA4 on microglial activation and TNF-α release.

## Discussion

Spinal cord injury triggers neuroinflammation and chronic pain. It is becoming increasingly apparent that controlling SCI-induced neuroinflammation can improve recovery from injury and neuropathic pain. However, few drugs have been shown to be safe and efficient in manipulating neuroinflammation and pain. Given the safety profile and pro-resolution and analgesic properties of the endogenous lipid mediators [13, 48–52], we investigated the potential anti-inflammatory and analgesic roles of the lipid mediator LXA4 in a rodent model of SCI. Our results clearly demonstrate that LXA4 effectively attenuates microglial reactivity and neuropathic pain after SCI.

We used an animal model of SCI consisting in thoracic spinal cord hemisection. Despite the partial nature of this type of spinal cord section, this model conserves several intact ascending and descending somatosensory pathways [22, 53], in which maladaptive plasticity results in neuropathic pain (i.e., mechanical allodynia in animal hind paws). This model is also characterized by the development of an extensive neuroinflammation around the injury site, which is also observed in patients suffering from SCI [54, 55].

Current analgesic treatments for SCI, such as anticonvulsants and antidepressants, target specific neuronal mechanisms and are mostly ineffective and accompanied by severe side effects [56, 57]. Here, we show that LXA4, an endogenous lipid mediator with no known toxicity, can alleviate SCI-induced neuropathic pain. Fatty acids derived from omega-3 and omega-6 have been previously involved in SCI [58–61] and DHA, in particular, shown to display promising analgesic effects [62]. Lipid mediators that are metabolized from fatty acids (i.e., resolvins, protectins, and lipoxins) generally have greater analgesic efficacy and potency in various animal models of inflammatory and neuropathic pain [7, 16, 17, 63, 64]. Indeed, we demonstrated that two intrathecal injections of LXA4 in the picomolar dose range were sufficient to alleviate mechanical allodynia for up to 28 days after SCI.

LXA4 mostly acts on the G protein-coupled ALX/FPR2 receptor [65, 66], but LXA4 can also bind to additional receptors, including the aryl hydrocarbon receptor, the cysteinyl leukotriene receptor, the GPR32 receptor, and the CB1 cannabinoid receptor [29, 67–70]. However, the finding that knockdown of ALX/FPR2 receptor expression with specific siRNA inhibited the analgesic effect of LXA4 indicates that this receptor is an important target for the effects of the lipid seen in SCI animals.

Our results also showed that LXA4 reduced the SCI-induced enhancement of reactivity of microglia and of expression of pro-inflammatory cytokines. Because spinal release of pro-inflammatory cytokines such as TNF­α, IL-6, and IL­1β are critical for the pathogenesis of pain [71–73], the anti-nociceptive effects of LXA4 could be attributable to its anti-inflammatory role.

After SCI, TNF-α levels become elevated in the spinal cord and may trigger pain hypersensitivity. Recently, we have demonstrated that microglial TNF-α can rapidly modulate synaptic transmission and pain hypersensitivity, including mechanical hyperalgesia [74]. TNF-α is a key pro-inflammatory pain mediator because it can influence multiple mechanisms involved in pain transmission. It can increase pre-synaptic pain transmission via activation of TRPV1 in C-type primary afferent terminals [74, 75] and also act on post-synaptic AMPA and NMDA receptors, increasing synaptic transmission in excitatory neurons [76, 77]. TNF-α can also decrease inhibitory synaptic transmission in the spinal cord [78, 79], stimulate the release of additional pro-inflammatory cytokines, and induce proliferation of immune and glial cells to enhance neuroinflammation and pain transmission [80]. For instance, TNF-α elicits astrocytic proliferation and release of pro-inflammatory and pronociceptive mediators [27]. Although we were unable to observe a diminishing effect of LXA4 treatment on the reactivity of astrocytes, one cannot exclude that LXA4 may act directly on astrocytes and reduce pro-inflammatory mediator release from these cells.

We showed the expression of the ALX/FPR2 receptor in primary microglial culture, and it has been previously demonstrated that LXA4 can also act on astrocytes inhibiting their activation and alleviating pain hypersensitivity in an animal model of inflammatory pain [15]. ALX/FPR2 receptors are found in a wide array of tissues, including the peripheral nervous system, macrophages, and neutrophils [28, 81, 82]. Thus, the beneficial effects of LXA4 after SCI should not be attributed solely to the regulation of astrocytes and microglia. However, we observed that LXA4 specifically decreased SCI-induced overexpression of microglial markers IBA-1 and P2Y12 in the spinal cord in vivo and inhibited phosphorylation of p38 and release of TNF-α in microglial culture stimulated by INF-γ, a commonly used activator of microglia. To support our data, the inhibition of microglial p-p38 by exogenous LXA4 has been recently reported in an animal model of hemorrhage [37]. In agreement with a role of microglia in SCI-induced neuropathic pain, a previous study has shown that spinal cord treatment with minocycline, a widely used microglial inhibitor, can attenuate the hyperresponsiveness of lumbar dorsal horn neurons and pain hypersensitivity following SCI [83].

Interestingly, we observed that late LXA4 treatment (at 35 days after SCI) is still effective in reducing neuropathic pain. A previous study showed that a treatment blocking TNF-α release effectively reduced SCI-induced mechanical hyperalgesia when given immediately after spinal cord hemisection but was ineffective when delivered 2–3 weeks after injury [84]. The analgesic effect of LXA4 in the late phase may thus be independent of TNF-α. Lipid mediators have multiple actions in addition to controlling neuroinflammation. They are also involved in the resolution of synaptic plasticity in the CNS and modulation of TRP channel activities [7]. Further studies will be needed to fully understand the mechanisms and appreciate the therapeutic potentials of LXA4 in spinal cord injury.

## Conclusions

Between 250 000 and 500 000 people become spinal cord injured every year, and most of them develop chronic pain. SCI is heterogeneous and characterized by neuronal apoptosis, demyelination, and neuroinflammation. Resident and invading inflammatory cells (including microglia) are associated with the development of persistent pain, and these immune cells can have either destructive or reparative roles after SCI. Collectively, our findings demonstrated that LXA4 could alter pro-inflammatory responses of microglia while sparing anti-inflammatory responses to SCI and, importantly, promote effective and persistent alleviation of mechanical allodynia. Given the potent anti-inflammatory, pro-resolution, and analgesic efficacy and safety profiles of LXA4, this endogenous lipid mediator may constitute a new therapeutic tool for the management of neuroinflammation and neuropathic pain following spinal cord injury.

## Additional files

Additional file 1: Figure S1. Lipoxin A4 reduces mechanical allodynia induced by spinal cord hemisection in rats. A, C, Following SCI, adult rats develop mechanical hypersensitivity in both ipsilateral and contralateral hindpaws. Frequency of responses is reduced with LXA4 administration, compared to vehicle-treated group. B, D, The area under the curve of frequency of paw withdrawal responses to mechanical stimulation of ipsilateral and contralateral paw over time. Results are presented as mean ± SEM. * denotes *p* < 0.05 when comparing to sham-operated group. # denotes *p* < 0.05 when comparing with vehicle-treated group (two-way ANOVA followed by Bonferroni; *n* = 8 rats/group). (TIF 2197 kb) Additional file 2: Figure S2. Lipoxin A4 reduces the expression of pro-inflammatory cytokines in the rat spinal cord. A–C, Spinal cord injury promotes a significant upregulation of IL-6, IL-1β, and TNF-α expression in the spinal cord. The three cytokines are significantly reduced by LXA4, in comparison to vehicle-treated group. D There is no significant change in the IL-10 cytokine levels. Results are presented as mean ± SEM. * denotes *p* < 0.05 when comparing to sham-operated group and # denotes *p* < 0.05 when comparing with vehicle-treated group (One-way ANOVA followed by Bonferroni; *n* = 4 rats/group). (TIF 1262 kb)

## Abbreviations

ALX/FPR2: formyl peptide receptor 2
DAPI: 4′,6-diamidino-2-phenylindole
GFAP: glial fibrillary acidic protein
Iba-1: ionized calcium-binding adaptor molecule 1
LXA4: lipoxin A4
SCI: spinal cord injury
siRNA: short-interfering RNA
