# Supplementary figures and images for: Lipoxin A4 inhibits microglial activation and reduces neuroinflammation and neuropathic pain after spinal cord hemisection
Source: J Neuroinflammation. 2016 Apr 8;13:75. doi: 10.1186/s12974-016-0540-8 (PMC4826542; doi:10.1186/s12974-016-0540-8)

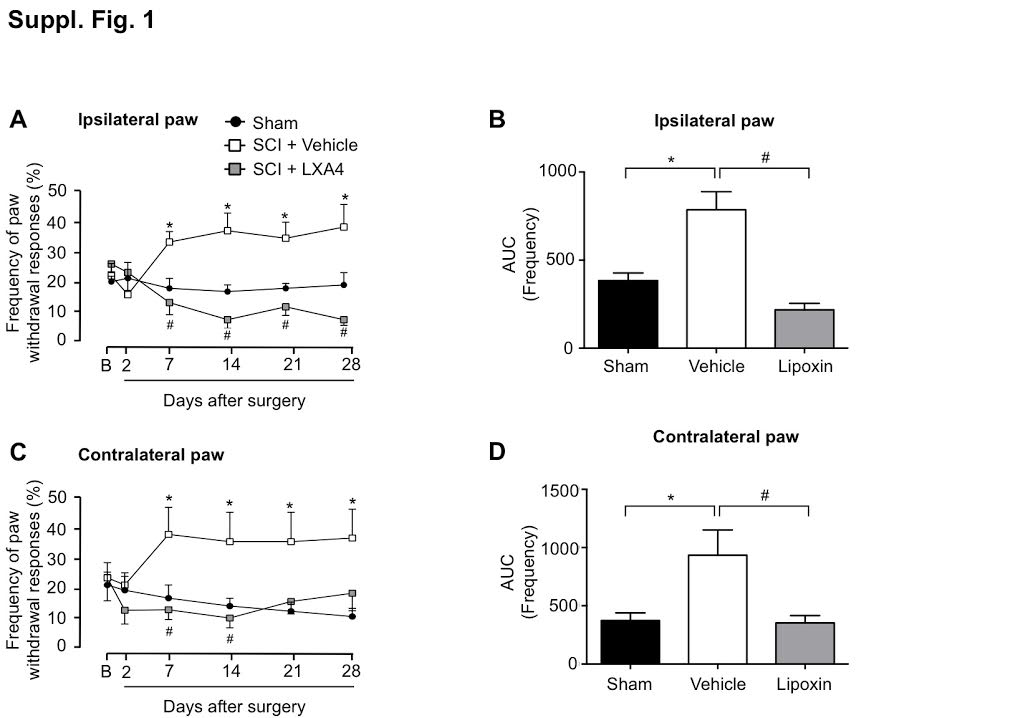

Supplement: Additional file 1: Figure S1. — Lipoxin A4 reduces mechanical allodynia induced by spinal cord hemisection in rats. A, C, Following SCI, adult rats develop mechanical hypersensitivity in both ipsilateral and contralateral hindpaws. Frequency of responses is reduced with LXA4 administration, compared to vehicle-treated group. B, D, The area under the curve of frequency of paw withdrawal responses to mechanical stimulation of ipsilateral and contralateral paw over time. Results are presented as mean ± SEM. * denotes p < 0.05 when comparing to sham-operated group. # denotes p < 0.05 when comparing with vehicle-treated group (two-way ANOVA followed by Bonferroni; n = 8 rats/group). (TIF 2197 kb) [file 12974_2016_540_MOESM1_ESM.tif]

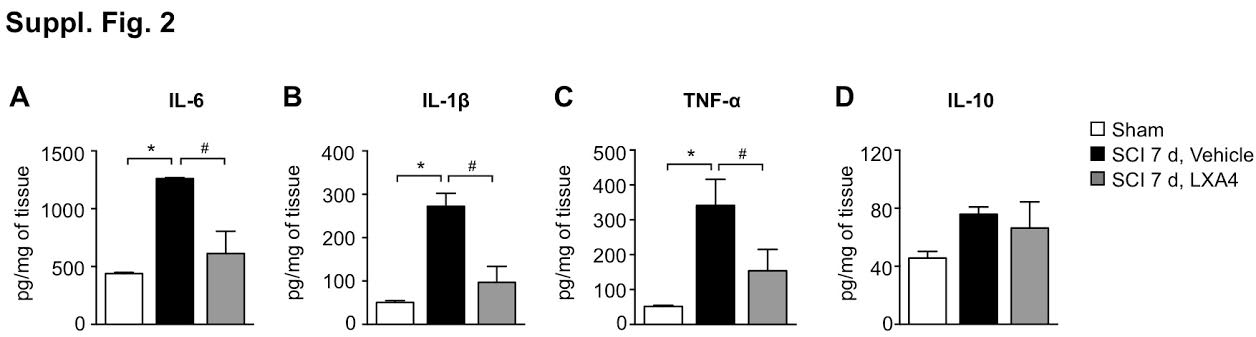

Supplement: Additional file 2: Figure S2. — Lipoxin A4 reduces the expression of pro-inflammatory cytokines in the rat spinal cord. A–C, Spinal cord injury promotes a significant upregulation of IL-6, IL-1β, and TNF-α expression in the spinal cord. The three cytokines are significantly reduced by LXA4, in comparison to vehicle-treated group. D There is no significant change in the IL-10 cytokine levels. Results are presented as mean ± SEM. * denotes p < 0.05 when comparing to sham-operated group and # denotes p < 0.05 when comparing with vehicle-treated group (One-way ANOVA followed by Bonferroni; n = 4 rats/group). (TIF 1262 kb) [file 12974_2016_540_MOESM2_ESM.tif]
